# Macrophage–Microbe Interactions: Lessons from the Zebrafish Model

**DOI:** 10.3389/fimmu.2017.01703

**Published:** 2017-12-01

**Authors:** Nagisa Yoshida, Eva-Maria Frickel, Serge Mostowy

**Affiliations:** ^1^Host-Toxoplasma Interaction Laboratory, The Francis Crick Institute, London, United Kingdom; ^2^Section of Microbiology, MRC Centre for Molecular Bacteriology and Infection, Imperial College London, London, United Kingdom

**Keywords:** host–pathogen interactions, infection, inflammation, macrophage, zebrafish

## Abstract

Macrophages provide front line defense against infections. The study of macrophage–microbe interplay is thus crucial for understanding pathogenesis and infection control. Zebrafish (*Danio rerio*) larvae provide a unique platform to study macrophage–microbe interactions *in vivo*, from the level of the single cell to the whole organism. Studies using zebrafish allow non-invasive, real-time visualization of macrophage recruitment and phagocytosis. Furthermore, the chemical and genetic tractability of zebrafish has been central to decipher the complex role of macrophages during infection. Here, we discuss the latest developments using zebrafish models of bacterial and fungal infection. We also review novel aspects of macrophage biology revealed by zebrafish, which can potentiate development of new therapeutic strategies for humans.

## Introduction

Macrophages are a major component of the innate immune system, responding efficiently to tissue damage and infection (1, 2). During infection, macrophages have diverse roles including phagocytosis of foreign bodies, release of cytotoxic factors, and coordination of the inflammatory response *via* the secretion of chemokines and cytokines (3, 4). Phagocytosis can involve the recognition of pathogen- and damage-associated molecular patterns (PAMPs and DAMPs, respectively) through pattern recognition receptors (PRRs) on the macrophage surface (5, 6). Further, the complement system can mark pathogens for phagocytosis by opsonization (7). Once internalized, the pathogen resides inside a vacuole known as a phagosome (8). Subsequent phagosome maturation involves acidification of the lumen, which leads to lysosomal fusion and degradation of the internalized microbe (9). Pathogen restriction is enhanced by the nutrient-limiting ability of the phagolysosome and the input of antimicrobial agents into the lumen, such as reactive oxygen/nitrogen species (ROS/RNS) (10). Although the majority of microbes succumb to the microbicidal environment within the phagolysosome, some pathogens (including *Mycobacterium tuberculosis* and *Salmonella Typhimurium*) can survive and replicate within this harsh environment (11, 12). In contrast, some bacterial pathogens (including *Listeria monocytogenes* and *Shigella flexneri*) have mechanisms to escape from the phagosome and proliferate in the cytosol (13).

Mechanisms of cell-autonomous immunity are crucial for protection of the host cell cytosol (14). Autophagy is an evolutionarily conserved process of intracellular degradation, recognized as an important defense mechanism against intracellular pathogens (15). Targeting of bacterial pathogens by the autophagy machinery is often mediated by ubiquitination, a posttranslational modification (16, 17). In this case, ubiquitinated substrates (such as bacterial components or damaged membrane) are recognized by autophagy receptors, including p62 and NDP52, which direct formation of the autophagic membrane around the targeted pathogen (18–20). Autophagy-related (ATG) proteins also direct immunity-related GTPases (IRGs) and guanylate-binding proteins (GBPs) to pathogens (21). IRGs and GBPs belong to a family of GTPases that confer host cell resistance during infection by pathogens (22–24). IRGs cooperate with GBPs to target non-self vacuoles, trafficking nicotinamide adenine dinucleotide phosphate (NADPH) oxidase, ATG proteins, and inflammasome complex assembly for host defense (25–27). Intracellular pathogens are also detected *via* nucleotide-binding oligomerization domain-like receptors (NLRs), a class of PRRs that reside in the cytosol (28). An important example is NLRP3, which acts as a scaffold protein for inflammasome assembly, leading to caspase-1 activation and maturation of pro-inflammatory cytokines interleukin-1β (IL-1β) and IL-18 (29, 30). These cytokines enhance the immune response and induce pathways leading to pyroptosis, a highly inflammatory type of programmed cell death (31).

A variety of different animal models have made important contributions to the study of macrophage-microbe interplay *in vivo*. Originally used for studying development, the zebrafish has many similarities with higher vertebrates (including mammals), which has led to their use for studying infection and immunity (32, 33). During the first 4 weeks of development, zebrafish lack adaptive immunity and rely on the innate immune response for host defense (34). This, together with *ex utero* development of embryos, necessitates the rapid development of innate immune cells, progenitors of which can be observed as early as 20 h postfertilization (hpf). Although the precise sites of development and maturation differ between zebrafish and human phagocytes, zebrafish macrophages retain close morphological and functional similarities with their mammalian counterparts (35). Primitive macrophages, originally identified by Philippe Herbomel and colleagues, use phagocytosis to control infection by *Escherichia coli* and *Bacillus subtilis* (36). Optical accessibility during early life stages make zebrafish larvae highly suited for non-invasive live microscopy. Studies on the zebrafish immune system identified macrophage-specific genes (including *mpeg1* and *csf1ra*), discoveries that enabled the development of specific reporter lines (37, 38). By using transgenic lines that fluorescently label distinct leukocyte populations, studies have identified key roles for macrophages during infection control *in vivo* (6). Furthermore, the sequenced zebrafish genome and the ability to manipulate the immune system through chemical or genetic means (including morpholino oligonucleotides for transient depletion, or CRISPR/Cas9 for genome engineering) make the zebrafish a unique and powerful tool for studying host–pathogen interactions at the molecular, cellular, and whole-animal level (39–41).

In this review, we discuss novel aspects of host–pathogen interactions that have been recently revealed using bacterial (mycobacteria, Gram-positive, Gram-negative) and fungal zebrafish infection models, highlighting key roles for macrophages in host defense against a variety of important pathogens.

## Zebrafish Macrophage–Bacteria Interactions *In Vivo*

**Figure 1:**
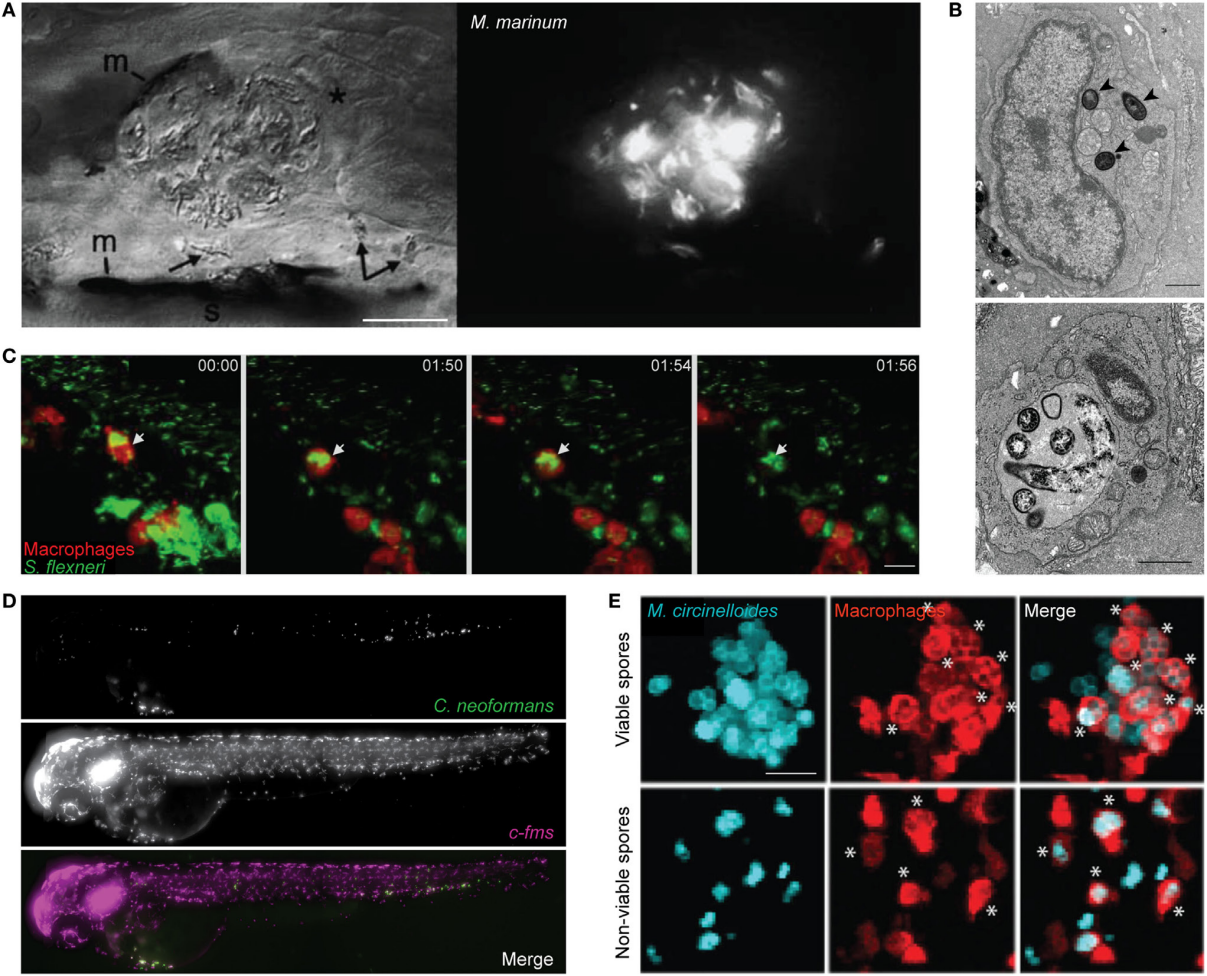
Zebrafish macrophage-microbe interactions *in vivo*. **(A)** Differential interference contrast (left) and fluorescent microscopy (right) image of macrophage aggregation to *Mycobacterium marinum* in the tail of wild-type AB larvae. Asterisk (*) indicates an infected macrophage at the aggregate; arrows indicate infected macrophages near the aggregate. m, melanocyte; s, striated muscle; scale bar 25 µm. Image adapted from Ref. (42). **(B)** Electron microscopy images of the caudal hematopoietic tissue of wild-type AB larvae injected intravenously with *Listeria monocytogenes* 3 h postinfection (hpi). *Listeria* in a macrophage cytosol (arrowheads; top image), and *Listeria* in a macrophage phagosome (bottom image). Scale bar 1 µm. Images adapted from Ref. (53). **(C)** Confocal time-lapse images of *Tg(mpeg1:G/U:nfsb-mCherry)* larva (red macrophages) infected with *Shigella flexneri* (green) by caudal vein injection, first frame at 20 min postinjection (mpi). White arrow depicts GFP-*Shigella* phagocytosed by a red macrophage, with a loss of red fluorescence at frame 01:56 indicating macrophage cell death. Maximum intensity projection of six planes every 2 µm, scale bar 10 µm. Images adapted from Ref. (54). **(D)** High content imaging of *Tg(fms:Gal4.VP16)il86; Tg(UAS:nfsb.mCherry)il49* larvae harboring macrophages (middle) injected with *Cryptococcus neoformans* (top) into the yolk sac circulation valley; bottom panel showing a merged image of both macrophages (magenta) and *C. neoformans* (green). Maximum intensity projection of images obtained 2hpi. Images adapted from Ref. (55). **(E)** Hindbrain ventricle injection of viable (top row) or non-viable (bottom row) *Mucor circinelloides* spores (cyan) in *Tg(mpeg1:G/U:nfsb-mCherry/mpx:GFP)* larvae harboring red macrophages imaged at 10 h 45 min and 1 h 5 min, respectively. Asterisks (*) indicate spores inside macrophages (red). Z-stack of 15 sections every 7.3 µm; scale bar 20 µm. Images adapted from Ref. (56). All adapted images were used with the appropriate permissions from the copyright holders of this work.

*Mycobacterium marinum* is a natural pathogen of zebrafish, closely related to the causative agent of human tuberculosis (*M. tuberculosis*). Pioneering studies have shown that *M. marinum* infection leads to the aggregation of macrophages into granuloma-like structures that both contain and promote bacterial dissemination (Figure 1A) (42, 43). These structures are initiated by the ESX-1 secretion system, which induces expression of matrix metalloproteinase-9 in epithelial cells to recruit macrophages for bacterial phagocytosis (44, 45). Tissue-resident macrophages first responding to infection are microbicidal. Therefore, to create a replication niche, *M. marinum* induces chemokine (C-C motif) ligand 2 (CCL2) expression and recruits uninfected monocytes *via* the surface lipid phenolic glycolipid (PGL) (46). Moreover, *M. marinum* possess cell surface-associated phthiocerol dimycoceroserate lipids, which impede PAMP–TLR interactions and prevent the microbicidal response in newly recruited monocytes (47). Consistent with a protective niche, the granuloma supports bacterial growth, alteration of granuloma structure through disruption of E-cadherin increases immune cell accessibility, and reduces bacterial burden (48). Work has shown that macrophage deficiency leads to accelerated necrosis of the granuloma and increased susceptibility to infection (45). A balanced inflammatory response is crucial to prevent necrosis of the granuloma, as both low and high levels of tumor necrosis factor (TNF) can lead to increased bacterial replication (49). Supporting this, a forward genetic screen performed in zebrafish revealed that mutation of the *lta4h* locus (encoding leukotriene A_4_ hydrolase) can modulate production of anti- and pro-inflammatory lipid mediators and susceptibility to mycobacterial infection (50). Angiogenesis has also been implicated in granuloma expansion and bacterial dissemination *via* a mechanism that requires hypoxia-induced vascular endothelial growth factor expression and C-X-C chemokine receptor type 4 (CXCR4) signaling (51, 52). Collectively, these studies highlight a complex role for macrophages in granuloma formation and in host defense against mycobacteria.

Infection by *Mycobacterium leprae*, an ancient pathogen that causes leprosy, is restricted to humans and nine-banded armadillos (*Dasypus novemcinctus*) (57). *M. leprae* infection causes demyelination of peripheral nerves and axonal damage, which can lead to symptoms such as muscle weakness and numbness. Remarkably, new work has shown that zebrafish can be used to study *M. leprae* pathogenesis *in vivo* (58). Although *M. leprae* replication is not observed in zebrafish due to its long doubling time of 12–15 days, the macrophage response to bacteria is comparable to that observed during *M. marinum* infection. In the case of *M. leprae*, PGL-1 is responsible for mediating structural changes in myelin by inducing macrophage RNS production, which subsequently causes mitochondrial swelling, demyelination, and axonal damage.

*Listeria monocytogenes*, a Gram-positive foodborne pathogen, can cause listeriosis and meningitis in immunocompromised individuals, and spontaneous abortions during pregnancy (59, 60). Inside macrophages, *L. monocytogenes* can escape from the phagosome and proliferate in the cytosol (Figure 1B) (61). Bacterial escape from the phagosome to the cytosol is linked to expression of listeriolysin O (LLO), a pore-forming toxin that targets the phagosomal membrane (62). ActA, another major virulence factor of *Listeria*, enables actin tail polymerization and autophagy escape (59, 63, 64). In agreement with studies performed *in vitro* using tissue culture cells, virulence of *Listeria* in zebrafish is dependent on LLO and ActA (53). More recent work using zebrafish has shown that bacterial dissemination (*via* necrosis of infected macrophages and release of bacterial-containing blebs) is LLO-dependent (65). To counteract this, Gp96 (an endoplasmic reticulum chaperone) can protect the integrity of the host cell plasma membrane against pore-forming toxins. In a separate study, zebrafish infection with a *Listeria* strain ectopically expressing flagellin (called *Lm*-pyro) was shown to activate the inflammasome in macrophage and reduce infection (66). These results highlight the inflammasome as crucial for protection against *Listeria*.

*Staphylococcus aureus* is an opportunistic pathogen, which latently resides in one-third of humans. Invasive surgery or lesions can increase the risk of infection, and considering the emergence of antibiotic-resistant strains, *S. aureus* is recognized as a major human threat. While systemic *S. aureus* infections are controlled, zebrafish are susceptible to yolk sac infection (i.e., a site inaccessible to leukocytes), underscoring the importance of leukocytes for infection control (67). Experiments performed using larvae depleted of myeloid cells demonstrate a role for macrophages in restriction of *S. aureus* proliferation in the blood (67). Interestingly, the incomplete clearance of bacteria by leukocytes can result in an “immunological bottleneck,” viewed to select for persisting bacterial populations (68). The zebrafish can, therefore, be used to discover mechanisms used by *S. aureus* to evade destruction within leukocytes.

Colonization of humans by *Burkholderia cenocepacia* has severe consequences in cystic fibrosis (CF) patients and other immunocompromised individuals. Originally viewed to form a biofilm in CF patients, studies using clinical samples have shown that *B*. *cenocepacia* can reside in alveolar macrophages (69). More recently, a zebrafish infection model demonstrated that macrophages are crucial for *B. cenocepacia* replication (70). Consistent with this, depletion of macrophages from larvae restricts bacterial replication (71). During infection, macrophages express IL-1β and recruit uninfected cells to form cellular aggregates (70, 71). Paradoxically, the depletion of IL-1β (using morpholino oligonucleotide) during *B. cenocepacia* infection results in decreased survival, yet, inhibition of IL-1β signaling (using the IL-1 receptor antagonist anakinra) results in increased survival (71). Together, these experiments suggest the precise role of IL-1β during *B. cenocepacia* infection, and its manipulation for therapy, is complex.

*Salmonella* is a well-studied Gram-negative pathogen responsible for gastroenteritis, enteric fever, and bacteremia. Investigation of *S. Typhimurium* has made important contributions to macrophage biology (6, 11). During zebrafish infection, *S. Typhimurium* can replicate within macrophages and also extracellularly within the vasculature (72). A subpopulation of intracellular bacteria is lysed by mitochondrial-derived ROS produced by macrophages *via* a pathway dependent on immunoresponsive gene 1 (IRG1) (73). In addition, macrophages are responsible for the “fine-tuning” of the immune response to *S. Typhimurium via* secretion of granulocyte-colony stimulating factor (G-CSF), which in turn stimulates the transcription factor C/ebpβ and enhances neutrophil production by emergency granulopoiesis (74).

*Shigella* is a Gram-negative enteroinvasive pathogen classified by the WHO as a global threat due to its development of antibiotic resistance (75–77). Among the species of *Shigella, S. flexneri* is best recognized as a paradigm for studying macrophage cell death (78). In agreement with studies performed *in vitro, S. flexneri* can induce cell death in zebrafish macrophages *in vivo* (Figure 1C) (54). Despite this, macrophage-depleted transgenic zebrafish present increased mortality during infection (79). These results suggest that macrophages play an important role in the initial collection of injected bacteria, prior to the elimination of bacteria and cellular debris by neutrophils. The increasing risk of multidrug-resistant bacteria has driven the need for treatments that do not strictly rely on antibiotics. Injection of *Shigella*-infected zebrafish with predatory bacteria *Bdellovibrio bacteriovorus* revealed a synergy between predator–prey interactions with the host immune system to restrict multidrug-resistant infection (80). In this case, the reduction of *Shigella* burden by *Bdellovibrio* is beneficial for infection control by zebrafish leukocytes.

## Zebrafish Macrophage–Fungus Interactions *In Vivo*

Invasive fungal infections are a growing problem, causing significant morbidity and mortality in organ transplant patients. Immunosuppression using calcineurin inhibitors is a common strategy for the prevention of organ transplant rejection and increases the risk of infection by *Aspergillus fumigatus* (81). Alveolar macrophages and inflammatory monocytes in the murine lung have been described as critical for early antifungal immunity during *Aspergillus* infection (82). Real-time visualization using a zebrafish infection model revealed the inability of neutrophils to phagocytose fungal spores, and suggested macrophages as crucial for host defense against *A. fumigatus* (83, 84). In mouse models of *A. fumigatus* infection, treatment with calcineurin inhibitor FK506 leads to increased mortality (85). Consistent with this, studies using zebrafish infection showed a role for calcineurin in protection against *Aspergillus* (86). In this case, calcineurin activation leads to dephosphorylation of nuclear factor of activated T cells (NFAT), and FK506 treatment impairs neutrophil recruitment because of reduced TNF-α production by macrophages (86). A separate study revealed FK506 inhibits the calcineurin-dependent lateral transfer of *A. fumigatus* from necroptotic to naïve macrophages, allowing fungal escape and unrestricted growth (87). Collectively, these studies highlight the indispensable role of calcineurin in macrophages for *Aspergillus* control *in vivo*.

*Candida albicans* is an opportunistic fungal pathogen, which primarily affects immunocompromised individuals. Zebrafish infection models have been used to identify *C. albicans* virulence factors and indicate an important role for the filamentous (hyphal) form of *C. albicans* in pathogenesis (88–90). Strikingly, real-time microscopy of *C. albicans* infection showed the extrusion of hyphae from the zebrafish hindbrain (88). Fungal dissemination is observed by 24 h postinfection (hpi), followed by lethality resulting from uncontrolled hyphal growth (89). Here, macrophages can restrict germination (but not replication), and fungal killing by macrophages and neutrophils is a rare occurrence (89). Zebrafish infection has also demonstrated a new role for NADPH oxidase in controlling hyphal growth, helping to recruit macrophages through ROS and preventing germination (89, 91).

Another opportunistic fungal pathogen, *Cryptococcus neoformans* can be fatal in immunocompromised individuals and is responsible for over 600,000 deaths globally per annum (92). Although highly informative, mammalian and non-vertebrate infection models have limitations in visualizing fungus-leukocyte dynamics and the translatability to higher vertebrate models, respectively. Live imaging of zebrafish during *Cryptococcus* infection revealed macrophages are required for pathogen control (Figure 1D) (55, 93). In agreement with this, macrophage depletion prior to infection leads to uncontrolled fungal replication and increased zebrafish mortality (55, 93). Macrophage-depletion postinfection also leads to increased fungal burden (55). The *Cryptococcus* capsule is made of polysaccharides contributing immunosuppressive functions, including the inhibition of phagocytosis. Consistent with this, capsule enlargement that occurs during infection of zebrafish can prevent phagocytosis, resulting in fungal proliferation and zebrafish mortality (55). By tracking individual macrophages over time, the first *in vivo* observation of vomocytosis (the controlled non-lytic expulsion of pathogens from phagocytes) was captured (55). The precise role of vomocytosis in host defense is not yet known.

*Mucor circinelloides* is an emerging fungal pathogen in which the incidence of infection is increasingly associated with aging populations (94). *M. circinelloides* causes mucormycosis, a disease with a wide range of symptoms including fever and gastrointestinal bleeding (95). Treatment of mucormycosis in humans remains costly and unsuccessful, and fatalities are often linked to corticosteroid treatment and immune defects (96, 97). In agreement with this, immunosuppression by corticosteroid treatment results in increased zebrafish mortality (56). Moreover, macrophage-depleted zebrafish succumb to infection, highlighting a key role for macrophages in *M. circinelloides* control. Remarkably, macrophages accumulate around viable spores in a manner similar to the granuloma structures described for *M. marinum* infection (Figure 1E) (56). While the role of macrophage clusters during *M. circinelloides* infection is not fully known, the zebrafish infection model can provide a novel platform to study macrophage–fungal interplay during mucormycosis.

## Discussion

Here, we describe recent mechanistic insights into the macrophage response to intracellular pathogens as revealed by zebrafish infection (Table 1). Although macrophage recruitment and phagocytosis is typically observed in response to infection, this is not always followed by pathogen restriction. Zebrafish infection has shown that, in some cases, macrophages can promote pathogenesis by shielding the pathogen from immune control or by providing a replicative niche. The zebrafish is a relatively new model for the study of human infectious disease. Therefore, a limitation of the system includes the lack of tools currently available, such as zebrafish antibodies and cell lines, which can impede in-depth mechanistic studies. On the other hand, the rapid development of transgenic zebrafish lines with fluorescently tagged proteins/cells, in combination with genome-editing technologies, compensate for these limitations. Considering advancements in RNAseq and high-resolution microscopy, we can expect that zebrafish infection will continue to illuminate fundamental aspects of host–pathogen interactions at the molecular, cellular, and whole animal level. The hope is that studying macrophage–microbe interactions *in vivo* using the zebrafish model can deliver therapeutic impact in humans.

**Table 1:** The macrophage response/role during zebrafish infection.

Microbe			Macrophage response/role	Reference
Bacteria	Mycobacteria	*Mycobacterium marinum*	Recruitment, phagocytosis, intracellular niche for bacteria, accumulation in granuloma-like structures, restriction and promotion of bacterial dissemination	(42, 45, 47, 48, 51, 52)
		*Mycobacterium leprae*	Recruitment, phagocytosis, RNS production causing axonal damage	(58)
	
	Gram-positive	*Listeria monocytogenes*	Recruitment, phagocytosis, intracytosolic niche for bacteria, restriction of bacterial dissemination	(53, 65, 66)
		*Staphylococcus aureus*	Recruitment, phagocytosis, restriction of bacterial proliferation in blood	(67, 68)
	
	Gram-negative	*Burkholderia cenocepacia*	Recruitment, phagocytosis, intracellular niche for bacteria, source of inflammatory response	(70, 71)
		*Salmonella Typhimurium*	Recruitment, phagocytosis, intracellular niche for bacteria, IRG1-dependent ROS production, stimulates emergency granulopoiesis by G-CSF secretion	(72–74)
		*Shigella flexneri*	Recruitment, phagocytosis, scavenging of bacteria prior to neutrophil control	(54, 79, 80)

Fungi		*Apergillus fumigatus*	Recruitment, phagocytosis, production of TNFα for neutrophil recruitment, lateral transfer of *Aspergillus* from necroptotic to naive macrophages	(83, 84, 86, 87)
		*Candida albicans*	Recruitment, phagocytosis, restriction of spore germination, rare fungal killing	(88, 89, 91)
		*Cryptococcus neoformans*	Recruitment, phagocytosis, expulsion of *Cryptococcus* by vomocytosis	(55, 93)
		*Mucor circelloides*	Recruitment, phagocytosis, accumulation around viable spores in granuloma-like structures	(56)

## Author Contributions

NY, E-MF, and SM jointly wrote the manuscript.

## Conflict of Interest Statement

The authors declare that the research was conducted in the absence of any commercial or financial relationships that could be construed as a potential conflict of interest.
